# Assessing Children’s Fine Motor Skills With Sensor-Augmented Toys: Machine Learning Approach

**DOI:** 10.2196/24237

**Published:** 2021-04-22

**Authors:** Annette Brons, Antoine de Schipper, Svetlana Mironcika, Huub Toussaint, Ben Schouten, Sander Bakkes, Ben Kröse

**Affiliations:** 1 Digital Life Center Amsterdam University of Applied Sciences Amsterdam Netherlands; 2 Department of Information and Computing Sciences Utrecht University Utrecht Netherlands; 3 Academy for Physical Education Amsterdam University of Applied Sciences Amsterdam Netherlands; 4 Play and Civic Media Amsterdam University of Applied Sciences Amsterdam Netherlands; 5 Department of Industrial Design Eindhoven University of Technology Eindhoven Netherlands; 6 Department of Computer Science University of Amsterdam Amsterdam Netherlands

**Keywords:** motor development, fine motor function, gamification, playful, motor skill assessment, Movement ABC (MABC), machine learning, motor function, motor skills, toys, children, game, movement assessment

## Abstract

**Background:**

Approximately 5%-10% of elementary school children show delayed development of fine motor skills. To address these problems, detection is required. Current assessment tools are time-consuming, require a trained supervisor, and are not motivating for children. Sensor-augmented toys and machine learning have been presented as possible solutions to address this problem.

**Objective:**

This study examines whether sensor-augmented toys can be used to assess children’s fine motor skills. The objectives were to (1) predict the outcome of the fine motor skill part of the Movement Assessment Battery for Children Second Edition (fine MABC-2) and (2) study the influence of the classification model, game, type of data, and level of difficulty of the game on the prediction.

**Methods:**

Children in elementary school (n=95, age 7.8 [SD 0.7] years) performed the fine MABC-2 and played 2 games with a sensor-augmented toy called “Futuro Cube.” The game “roadrunner” focused on speed while the game “maze” focused on precision. Each game had several levels of difficulty. While playing, both sensor and game data were collected. Four supervised machine learning classifiers were trained with these data to predict the fine MABC-2 outcome: k-nearest neighbor (KNN), logistic regression (LR), decision tree (DT), and support vector machine (SVM). First, we compared the performances of the games and classifiers. Subsequently, we compared the levels of difficulty and types of data for the classifier and game that performed best on accuracy and F1 score. For all statistical tests, we used α=.05.

**Results:**

The highest achieved mean accuracy (0.76) was achieved with the DT classifier that was trained on both sensor and game data obtained from playing the easiest and the hardest level of the roadrunner game. Significant differences in performance were found in the accuracy scores between data obtained from the roadrunner and maze games (DT, *P*=.03; KNN, *P*=.01; LR, *P*=.02; SVM, *P*=.04). No significant differences in performance were found in the accuracy scores between the best performing classifier and the other 3 classifiers for both the roadrunner game (DT vs KNN, *P*=.42; DT vs LR, *P*=.35; DT vs SVM, *P*=.08) and the maze game (DT vs KNN, *P*=.15; DT vs LR, *P*=.62; DT vs SVM, *P*=.26). The accuracy of only the best performing level of difficulty (combination of the easiest and hardest level) achieved with the DT classifier trained with sensor and game data obtained from the roadrunner game was significantly better than the combination of the easiest and middle level (*P*=.046).

**Conclusions:**

The results of our study show that sensor-augmented toys can efficiently predict the fine MABC-2 scores for children in elementary school. Selecting the game type (focusing on speed or precision) and data type (sensor or game data) is more important for determining the performance than selecting the machine learning classifier or level of difficulty.

## Introduction

### Background

Motor development is crucial in child development. Acquiring motor skills is not only essential for daily life functioning but also influences children’s cognitive and social development [1]. Fine motor skills are a strong predictor of school results [2]. Motor skill development is not a fixed linear process. Every child has his/her unique learning curve and pace, and their motor skills develop by leaps and bounds [3]. Because of this unique and unpredictable motor development path, it is important to monitor children’s motor development over time instead of assessing them once. That way, insight in the progress of their motor development can be given [3,4]. Children with fine motor development problems have difficulties with learning fine motor skills. They experience, for instance, problems with school tasks such as writing or cutting or daily life activities such as closing a zipper or tying shoelaces [4]. In total, 5%-10% of children in elementary school have developmental motor problems [5,6]. When monitoring children’s motor development over time, these motor development problems can be recognized at an early stage. Consequently, appropriate diagnostic methods and required therapy could be started in time, which may diminish the effects of their motor development problems.

Both worldwide and in the Netherlands, the Movement Assessment Battery for Children Second Edition (MABC-2) is the major test for assessing children’s motor development [5,7]. The MABC-2 consists of both tests for fine and gross motor skills. Finishing the fine motor skill part of the MABC-2 (fine MABC-2) takes approximately 15 minutes per child and requires a trained supervisor [7]. Elementary school would be a natural place to test children’s motor skills since developmental motor problems affect cognitive development and school results and vice versa. Proficient fine motor skills are, for instance, essential for children to learn handwriting [8]. However, teachers in elementary school report that they do not have the required expertise and time to test all children, let alone to monitor them all over time.

Sensor-augmented toys and machine learning have been presented as possible solutions for the problems that teachers experience with the current assessment methods [9,10]. Both sensor data, regarding movements made with the toy, and game data, regarding events that occur in the game, can be collected while playing. Such a sensor-augmented toy can, for instance, measure the smoothness of movements made with the toy or how accurately a game was played. These data can be used to train machine learning algorithms in predicting children’s motor skill levels. After training and testing those machine learning algorithms, they can be used to classify whether children have fine motor development problems or not. Thus, sensor-augmented toys can be used as an assessment tool for signaling fine motor development problems in children.

Using sensor-augmented toys for indicating fine motor development problems in children has many advantages. First, these toys do not require a trained supervisor and require less instruction time. Moreover, such toys provide more secure data collection compared to manually collected data. Furthermore, playing games can safely be considered to be more enjoyable for children than standard assessment methods. Last, since no trained supervisor is required and children can easily play with the toys in the classroom, the toys enable testing in a natural setting instead of a testing environment. By playing games in such a natural setting, the assessment can be kept implicit and, therefore, children are not aware of undergoing the assessment [11].

### Related Work

Despite the advantages of evaluating children’s fine motor skills with sensor-augmented toys and machine learning, limited research is done in this field. Gamification of assessment processes in other contexts such as cognitive assessments has been studied before [12]. The systematic review of Lumsden et al [12] shows that many gamified cognitive assessments have been validated successfully. Although gamification in the field of health and well-being is popular, most studies focus on promoting physical activity levels, mental health, and people with a chronic disease [13,14]. Only a few studied gamification in the context of motor skills and most of them involved training instead of assessment [15,16]. Moreover, most of those studies involved patients with motor problems that were primarily caused by medical conditions such as cerebral palsy or stroke. Those children are already seen by medical specialists who monitor their motor development. In contrast, our study involved children who may have a delay in their motor skill development but do not have such diseases.

To the best of our knowledge, only 3 studies involved smart toys for assessing children’s fine motor skills [17-19]. Vega-Barbas et al [17] only performed a usability and feasibility test with smart toys that are potentially helpful for assessing motor skills, but they have not used it for assessment yet. The remaining 2 studies did use toys to evaluate children’s fine motor skill levels, but both involved toddlers instead of elementary school children. Moreover, they did not build a classification model that might predict the outcome of current motor skill assessment tests. Rivera et al [18] studied the intraindividual variability. Guitiérrez García et al [19] did build a regression model, but this model has not been tested and used to classify the fine motor skill level based on the sensor data yet. In addition to the sensor data that both studies included, we will also include game data, that is, data about events that occur in the game, to study its additional value.

In preliminary research, we studied the possibilities to use sensor-augmented toys for fine motor skill assessment [9,10]. We studied whether a toy called the Futuro Cube could be used to predict the outcome of the fine MABC-2 [9]. While a game was played with the toy, information regarding events occurring in the game was registered. In addition to these game data, sensor data were collected by measuring the movements of the toy with accelerometers inside the toy. These sensor data and game data were used as input for several supervised machine learning models, which then were used to classify the motor skill level of children. Our previous study showed that a machine learning model that uses sensor and game data of the Futuro Cube as input has the potential to classify the fine motor skill levels of children aged 7-8 years.

### Objective of This Study

In this study, in which a larger number of children participated, we improved our toy and game compared to that used in our preliminary research. First, we explored whether additional sensor features would improve the results. Second, we studied a game in which children could choose their own pace in the game since such elements are also included in the fine MABC-2. Therefore, we added a gyroscope to the toy to collect rotational data in addition to acceleration data. Moreover, we designed an additional game that focusses on precision instead of speed. Based on these modifications, we will answer the following research question: *What is the influence of the classification model, game, type of data, and level of difficulty (LoD) on predicting the fine MABC-2 scores for children aged 6-9 years with playing games with the Futuro Cube?*

## Methods

### Recruitment

Children were recruited through their elementary school teachers. Since we required a sufficient number of participants having motor development problems for balanced class labels, we included 2 elementary schools in Amsterdam that were known for having a larger population of children with motor development problems. Children who were between the age of 6 and 9 years and who were in the 3rd or 4th year of elementary school were included. Fine motor development is important for handwriting education. In the Netherlands, handwriting education starts in the 3rd year of elementary school and is a very important part of the 4th year of elementary school. Teachers of those classes reported that they need to know whether children’s fine motor development is proficient to start such education. Therefore, we chose not to include children in classes higher than the 4th year of elementary school. Pilot tests of our game showed that the explanation of the game was too hard for some children younger than 6 years. To make sure that all children understood how to play games with the toy, we chose not to include children younger than 6 years. Written informed consent was obtained from parents or legal guardians for participation of the child. A separate informed consent was acquired for publication of the pseudonymized raw data. In total, written informed consent for participation was given for 99 children and written informed consent for publication of the raw data was given for 49 children. A pseudonymized data set consisting of the sensor and game data of these children and their corresponding fine MABC-2 scores is available on request from the corresponding author. This study was performed according to the Declaration of Helsinki [20].

### Procedures

#### Test Setup

Each child was tested for 25 minutes. The fine MABC-2 was taken in 15 minutes by a trained supervisor. Further, the child played 2 different games on the Futuro Cube. Each game started with a short instruction, followed by a warming-up phase in which the child was able to become familiar with the game. Half of the participants started with the fine MABC-2 and subsequently played with the toy. For the other half of the participants, the order was the other way around: first playing with the toy and subsequently performing the fine MABC-2.

#### Determining the Level of Fine Motor Skills

The subscale for the measurement of fine motor skills of the MABC-2 was used to determine the fine motor skill level of the children. The fine MABC-2 test consists of 3 subtests. In the first subtest, children had to place 12 pegs in a board with 12 holes. In the second subtest, children had to thread a lace back and forth through a lacing board with holes. Both the first and second subtests were time-sensitive. The child was told to perform the task as quickly as possible and the time to complete the task was denoted as the raw score of those subtests. In the last subtest, children had to draw a trail with a pencil. They had to draw a single line and were not allowed to cross the trail’s boundaries. This subtest was not time-sensitive and the raw score consisted of the number of errors, that is, the number of times that the drawn line crossed the boundaries [7]. The raw scores of each subtest were summed to a raw total score. Based on the age of the participant, the raw total score was converted to a percentile score. This score, between 0 and 100, indicates the fine motor skill level of the participant compared to that of the children within the same age band. The higher the score, the better the fine motor performance of the participant compared to children of the same age. According to the MABC manual, a score in the 16th percentile or lower was defined as likely to have fine motor development problems. All scores in the 17th percentile or above were defined as not having fine motor development problems.

#### Toy and Games

**Figure 1:**
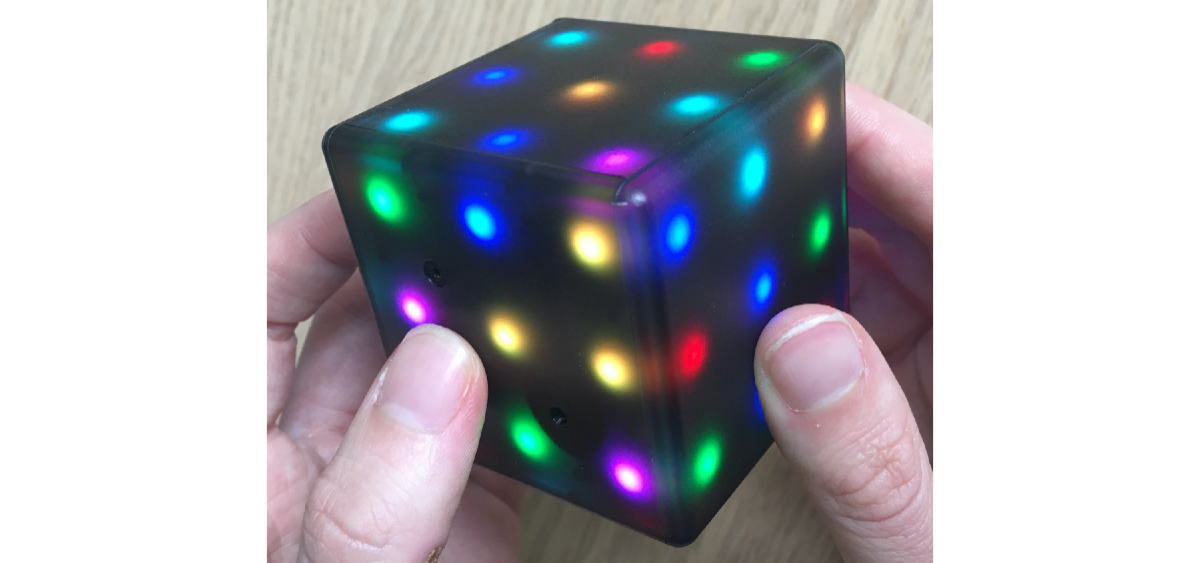
Futuro cube.

The Futuro Cube, which is shown in Figure 1, is a commercial toy that was adapted for research [21]. The cube has 9 colored lights on each side. The accelerometer and gyroscope inside the cube track motion, sense rotation, and measure orientation. The cube is 52×52×52 cm with 9 colored light-emitting diodes (LEDs) on each side. Each square can be identified by a unique index number i ∈ {0, …, 53}. This index number can be used to register the activation of an LED, including the color. The toy contains a tri-axial accelerometer with an acceleration sensitivity of ±8G and a tri-axial gyroscope with an angular rate sensitivity of 2000 dps. Based on these inertial measurement unit sensors, the orientation and the change of position can be recorded. Data collected with these sensors will be referred to as sensor data. The programming language PAWN2 was used for creating the games that are played with the toy. In the programmed script, we defined which information about events that occur during a game should be saved, for example, the change of color of an LED during the game. Saved data about the game will from now on be referred to as game data. While playing, both the game data and the abovementioned sensor data were registered. Bluetooth low energy was used to wirelessly send all data in real time from the cube to a computer with a sample frequency of 110 Hz. In both games, a highlighted dot was moving on the cube’s surface by activating the colored LEDs. In the first game, called the roadrunner game, the focus was on speed. The second game, called the maze game, focused on precision. In both games, no points were collected and neither visual nor auditory feedback was given about how well the game was played. In the roadrunner game, the velocity of the moving dot was predetermined and the child had to follow this speed. In the second game, however, the child was asked to move the dot as precisely as possible through the path without being tied to a certain pace.

**Figure 2:**
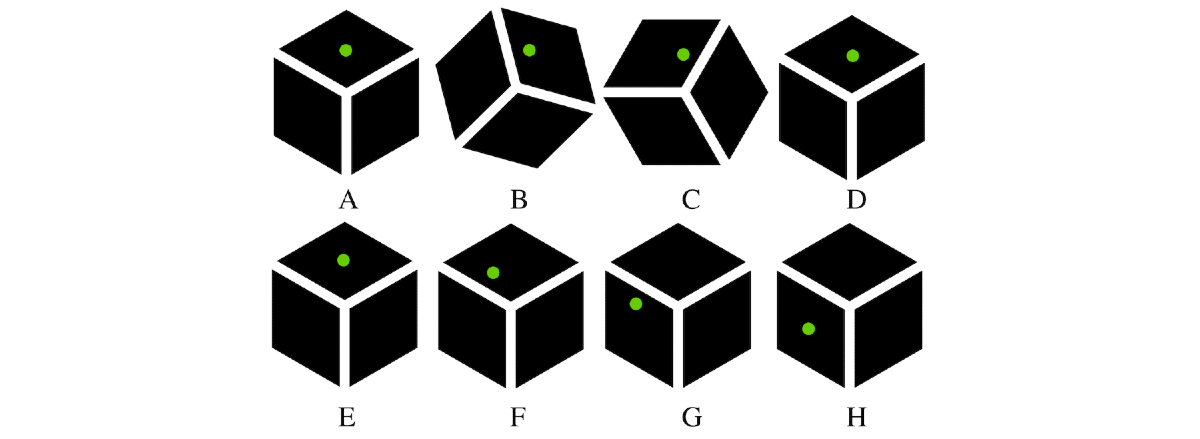
Schematic overview of the roadrunner game. A-D: show the way the cube should be rotated to keep the green dot in the correct position. E-H: show what happens in case the cube was not rotated at all.

In the roadrunner game, a green dot moved on the surface of the cube. The player was asked to rotate the cube in order to keep the spot on the top surface of the cube, which is shown in Figure 2. The dot moved with a certain velocity on the cube surface jumping from LED to LED. In case the spot was at the center LED of a side, it randomly turned left or right or kept moving forward. The velocity at which the spot moved was defined as the LoD. This game had 3 LoDs: LoD ∈ {0,1,2}. The lower the level, the longer the spot remained at the same place. Thus, level 0 was the easiest level and level 2 was the hardest level. The time that the spot remained at the same index number was denoted as the delay in seconds. The LoDs correspond to a delay *d* ∈ {0.8, 0.6, 0.4}. Each level lasted for 30 seconds and occurred twice. Hereby, it has to be taken into account that each player started with the easiest level and 2 subsequent levels could not have the same LoD. The order of the LoDs was randomized and the game started with a warming up phase of 60 seconds to discover the game. Table 1 shows all 10 possible permutations along with their order of LoDs.

**Table 1:** All possible permutations for the roadrunner game.

Permutation	Order of levels of difficulty	Participants (n)
0	0, 1, 2, 0, 1, 2	10
1	0, 1, 2, 0, 2, 1	10
2	0, 1, 2, 1, 2, 0	10
3	0, 1, 2, 1, 0, 2	10
4	0, 2, 1, 0, 1, 2	10
5	0, 2, 1, 0, 2, 1	9
6	0, 2, 1, 2, 0, 1	10
7	0, 2, 1, 2, 1, 0	10
8	0, 1, 0, 2, 1, 2	7
9	0, 2, 0, 1, 2, 1	9

**Figure 3:**
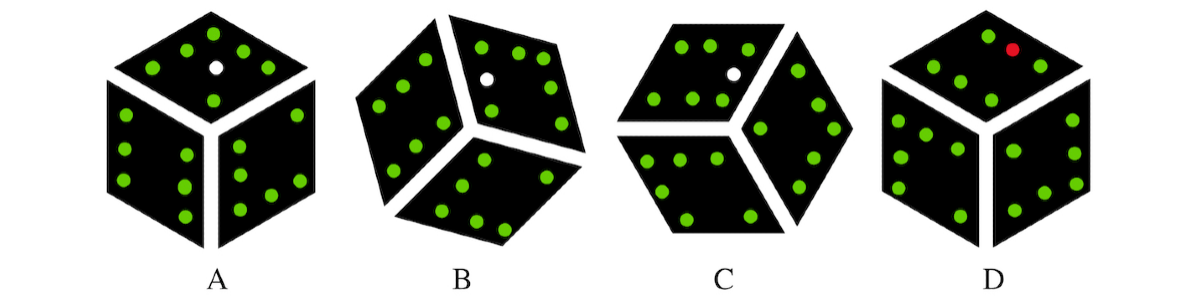
Schematic overview of the maze game. A-C: show the way the cube should be rotated to correctly move the white dot through the maze. D: shows what happens in case the white dot was moved to a location wherein it was not allowed to enter.

**Figure 4:**
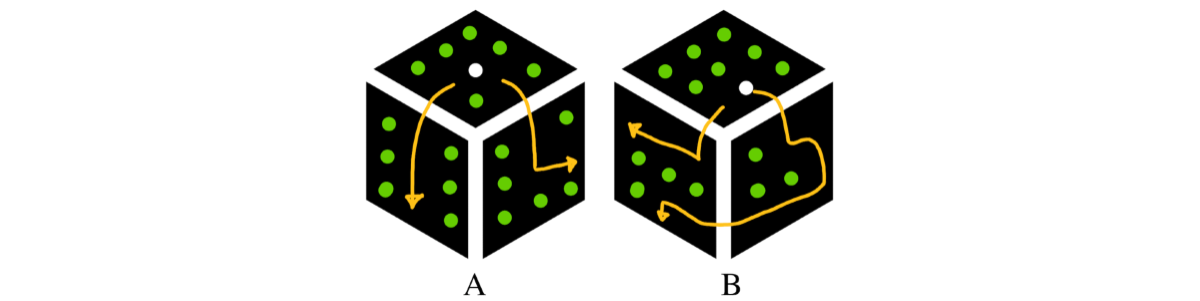
Schematic overview of the levels of the maze game. A: easiest level (level 0); B: hardest level (level 1).

The goal of the maze game was to move a white dot through a maze of green dots, as is shown in Figure 3. The dot could be moved by rotating the cube. In case the player moved the white dot on a location that was green and thus not allowed to enter, the dot turned red. When the player moved the white dot back on the right track, the red dot returned green. The path that the players were asked to walk through with the white spot was created by displaying green LEDs on the cube at certain index numbers. The game had 2 different levels of difficulty, indicated by the index numbers that turned green and thus were not allowed to enter with the white dot: LoD ∈ {0, 1}. Level 0 was the easiest level. Here, the player only had to push the white dot around the corner of the toy in the middle of a side. In the hardest level, pushing around the corner of the toy could also have to take place at the edges of the cube. A schematic overview of both levels of the maze game is shown in Figure 4. The path in the maze was infinite and each level lasted for 60 seconds. Similar to the roadrunner game, each level occurred twice and the real levels were preceded to a warming up phase of 60 seconds. No permutations could be made to randomize the LoDs since there were only 2 LoDs; each game started with the easiest level and 2 subsequent levels could not have the same LoD.

### Features

Before constructing features from the sensor data, we filtered the raw sensor data. Both the acceleration data and gyroscope data were filtered with a low-pass filter with a cut-off frequency of 4 Hz. The so-called feature jerk was calculated as the derivative of the acceleration and indicates the smoothness of the translational movements. To indicate the smoothness of the rotational movements, the derivative of the angular velocity was calculated. For each game, we have built 1 game feature. The game feature for the roadrunner game is the cosine similarity. This feature indicates how accurately a player kept the green moving dot on the top surface of the cube. The cosine similarity indicates the similarity between the location of the dot and the location of the top of the cube. The cosine similarity has a range of –1 to 1, with –1 meaning opposite orientations and 1 meaning identical orientations. Thus, for the roadrunner game, a cosine similarity value of 1 indicates that the player kept the cube exactly in the preferred position regarding the location of the green dot. For the maze game, the game state feature is the maze correctness. This feature indicates the time that the white dot was on the correct path.

We added gender and age of the participant as general features to the sensor and game features. Table 2 shows all the features together with its meaning in the context, which game the feature applies to, and what type of feature it is. For each game, the data of the warming-up phase (ie, the first 60 seconds of each game) were removed since the warming up was intended to familiarize the participant with the toy and was not part of the assessment. The mean value of a variable over time (eg, mean acceleration, mean jerk) and standard deviation were calculated for each sensor and game feature. Thus, we constructed 8 sensor features, 2 game features, and 2 general features per game.

**Table 2:** Overview of all the features.

Feature name	Meaning in context	Game	Type of feature
a	Total acceleration (m/s^2^)	Both	Sensor
ω	Total angular velocity (rad/s)	Both	Sensor
Jerk	Smoothness of the translational movements, derivative of a	Both	Sensor
α	Smoothness of the rotational movements, derivative of ω	Both	Sensor
Cosine similarity	Accuracy of keeping the green dot at the preferred position	Roadrunner	Game
Maze correctness	Time being on the correct path	Maze	Game
Gender	Gender	Both	General
Age	Age in years	Both	General

### Classification Models

Four different supervised machine learning algorithms were compared: k-nearest neighbor (KNN), logistic regression (LR), decision tree (DT), and support vector machine (SVM). LR and DT were selected because they provide interpretable models, which is important for teachers who will use the toy in their classrooms. KNN and SVM were chosen because they are known to have good performance with nontextual data, of which SVM often performs well with relatively little data [22]. The labels that were used for training and testing the classification model were the binary outcomes of the fine MABC-2. A child who was likely to have fine motor skill development problems according to the fine MABC-2 was denoted as 1, while a child not having fine motor skill development problems according to the fine MABC-2 was labeled as 0. The performance of the classification model was analyzed with stratified 49-fold cross validation [23]. Since our data set is relatively small, we chose to perform cross validation to prevent overfitting. Because the data set included 49 children with label 1, we performed 49-fold cross validation to maximize the use of the available data. Ideally, the distribution of class labels is almost equal in the training and test set. Stratified cross-validation enables this ideal distribution while performing cross validation [24]. Since our data set consisted of approximately as many children with label 1 as children with label 0, each test set of a fold consisted of one child with label 1 and one child with label 0. The label of each child was used as a test set in at least one fold because we performed 49-fold cross validation and the data set contained 49 children with label 1. Since the data set included 46 children with label 0, the data of 3 children were reused in the test set of another fold to enable stratified cross-validation.

### Comparing Performances

In the first analysis, the games and classifiers were compared. We trained and tested 4 different machine learning algorithms on all features of the roadrunner game and all features of the maze game. We used accuracy to indicate the percentage of the correctly classified cases. As an additional performance metric, we used the F1 score since it considers both precision and recall and these are both important in an assessment tool. Precision indicates the proportion of cases labeled positive that were actually correct, whereas recall indicates the proportion of actual positive cases that were labeled correctly. Wilcoxon tests were performed to show whether the best performing classifier performed significantly better than the other 3 classifiers. Moreover, Wilcoxon tests were performed to show the differences between the roadrunner game and the maze game for each classifier. For all statistical tests, we used α=.05. The game and classifier that performed best were used in the second analysis. Here, we compared the LoDs and the type of input features. For each possible combination of LoDs of the roadrunner game, we trained and tested the best performing classifier on the sensor features, the game features, and both sensor and game features. The general features such as age and gender were always included as classifier input. Wilcoxon tests were performed to show whether the best performing combination of the levels performed significantly better than the other combinations of levels. Furthermore, Wilcoxon tests were performed to show whether there were differences between the type of features for the best performing combination of levels.

## Results

### Participant Characteristics

In total, 95 children (52 girls and 43 boys) participated. Their mean age was 7.8 (SD 0.7) years. Based on the fine MABC-2 scores, 49 children showed problems with their fine motor skills, while 46 children did not have fine motor skill problems. Thus, the group of children having fine motor skill problems according to the fine MABC-2 score and the group of children not having fine motor skill problems were almost equal in number. Since we deliberately included urban elementary schools having a larger population of children with motor skill problems, this ratio is higher than the typical percentage of 5%-10% of the children having motor skill problems [5,6].

### Effects of the Games and Classifiers

**Figure 5:**
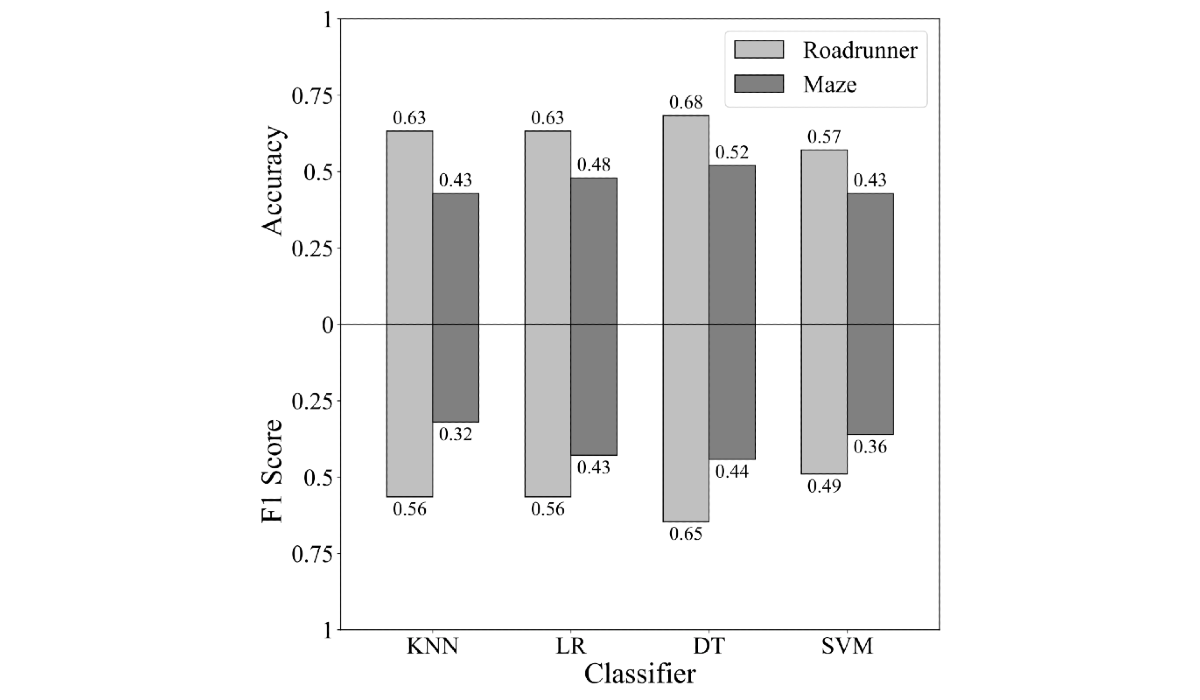
Mean accuracy and mean F1 scores for the comparison of the classifiers and games. DT: decision tree; KNN: k-nearest neighbor; LR: logistic regression; SVM: support vector machine.

The DT classifier with only features of the roadrunner game as input performed best with a mean accuracy score of 0.68 and a mean F1 score of 0.65. The corresponding mean recall score was 0.74. The DT classifier also performed best for the maze game with a mean accuracy score of 0.52, a mean F1 score of 0.44, and a mean recall score of 0.51. For all classifiers, the highest mean accuracy and mean F1 scores were achieved with features of the roadrunner game used as input. An overview of all mean accuracy and mean F1 scores per game and machine learning algorithm is shown in Figure 5.

Although the DT classifier achieved higher accuracy and F1 scores than the other 3 classifiers for both the roadrunner and the maze games, no significant differences were found. All results of the performed Wilcoxon tests to study differences between the DT and the other classifiers are shown in Table 3.

**Table 3:** Results of the Wilcoxon tests when the performance scores of the decision tree classifier were compared with those of the other classifiers.

Classifier	Accuracy (*P* value)		F1 score (*P* value)	
	Roadrunner game	Maze game	Roadrunner game	Maze game
k-nearest neighbor	.42	.15	.41	.14
Logistic regression	.35	.62	.18	.90
Support vector machine	.08	.26	.08	.23

Each classifier performed significantly better on accuracy with data obtained from playing the roadrunner game than with that obtained from playing the maze game (DT, *P*=.03; KNN, *P*=.01; LR, *P*=.02; SVM, *P*=.04). Except for the SVM classifier, each classifier also performed significantly better on the F1 score with data obtained from playing the roadrunner game than with data obtained from playing the maze game (DT, *P*=.02; KNN, *P*=.01; LR, *P*=.049). Table 4 shows all the results of the performed Wilcoxon tests to show differences between data obtained from playing the roadrunner game and the maze game.

**Table 4:** Results of the Wilcoxon tests when the performance scores of the roadrunner game were compared with those of the maze game for all classifiers.

Classifier	Accuracy (*P* value)	F1 score (*P* value)
Decision tree	.03^a^	.02^a^
k-nearest neighbor	.01^a^	.01^a^
Logistic regression	.02^a^	.049^a^
Support vector machine	.04^a^	.20

^a^Differences were statistically significant at *P*<.05.

### Influence of the Game Levels and Features

**Figure 6:**
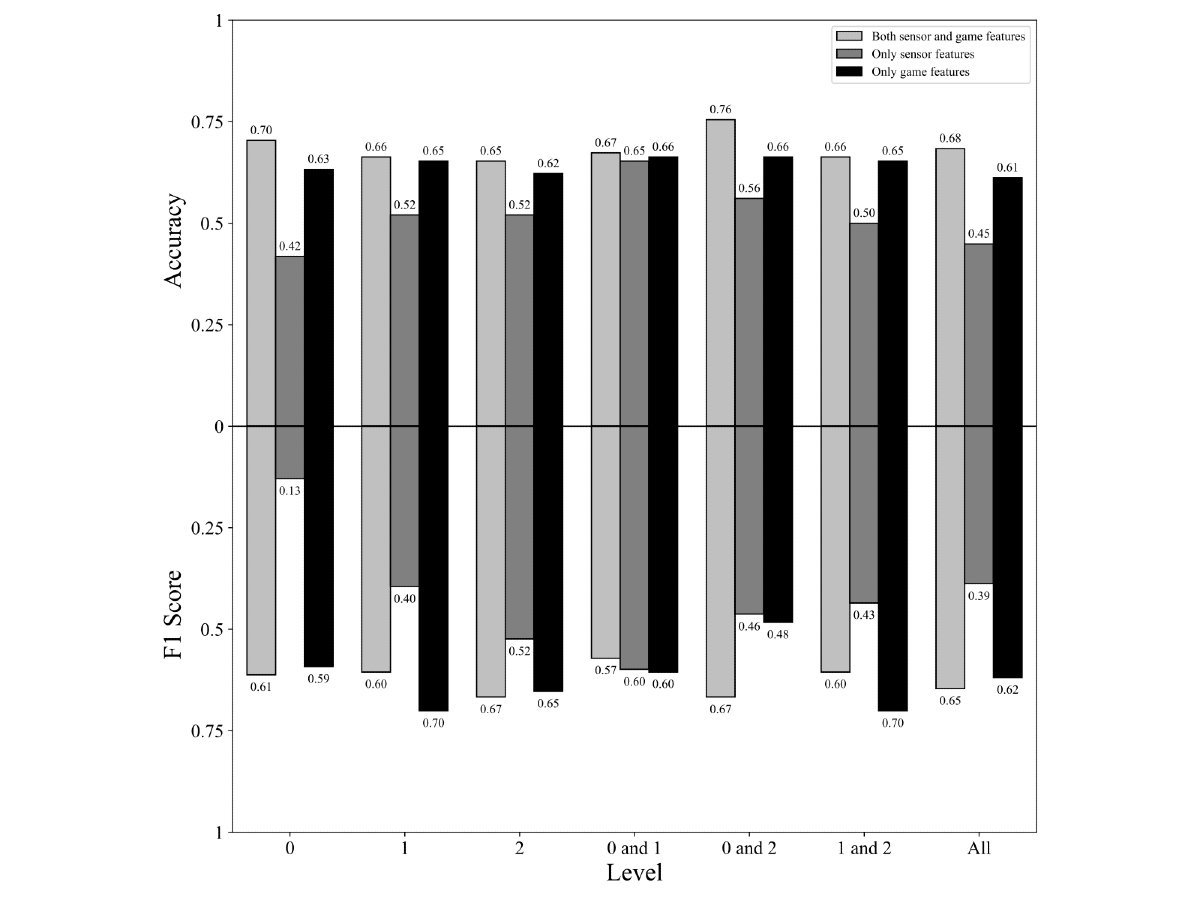
Mean accuracy and F1 scores for the comparison of the levels and types of input features.

The DT classifier with features of only the roadrunner game as input was used to compare the types of input features and combinations of levels since the combination of this classifier and game performed best in the first analysis. The highest mean accuracy, being 0.76, was achieved with a combination of data obtained from playing level 0 and level 2 and a combination of both sensor and game features. The corresponding mean F1 and recall scores were 0.67 and 0.71, respectively. The best mean F1 score, being 0.70, was achieved with the combination of level 1 and level 2 and only using game features. The corresponding mean accuracy and recall scores were 0.65 and 0.80, respectively. Figure 6 shows an overview of all mean accuracy and mean F1 scores per combination of levels and type of input features achieved with the DT classifier and data of the roadrunner game. Since the combination of data obtained from playing levels 0 and 2 with both sensor and game features achieved the highest mean accuracy, we compared levels 0 and 2 with the other combinations of levels. The combination of level 0 and level 2 only performed significantly better than the combination of level 0 and level 1 when both sensor and game features were used as input (*P*=.046). All results of the performed Wilcoxon tests to study differences between the combination of levels with both sensor and game features used as input are shown in Table 5.

**Table 5:** Results of the Wilcoxon tests when the performance scores of game level 0 and level 2 were compared with those of the other levels.^a^

Game level	Accuracy (*P* value)	F1 score (*P* value)
Level 0	.38	.54
Level 1	.21	.52
Level 2	.11	.92
Level 0 + 1	.046^b^	.10
Level 1 + 2	.21	.52
Level 0 + 1 + 2	.20	.81

^a^In all cases, decision tree was used as the classifier and both sensor and game features of the roadrunner game were used as input.

^b^Differences were statistically significant at *P*<.05.

For all combinations of levels, the combination of both sensor and game features performed better regarding accuracy than only one of those features. When zooming in on the best performing combination of levels, that is, level 0 and level 2, we found a significant difference in the accuracy and F1 scores between using both types of features and using only sensor features (accuracy, *P*=.001; F1 score, *P*=.01). The results of the performed Wilcoxon tests to study differences between the type of input features for the combination of level 0 and level 2 are shown in Table 6.

**Table 6:** Results of the Wilcoxon tests when the performance scores of the types of input features were compared.^a^

Input feature	Accuracy (*P* value)	F1 score (*P* value)
Both features versus only sensor features	.001^b^	.01^b^
Both features versus only game features	.18	.06
Sensor versus game	.10	.91

^a^In all cases, decision tree was used as the classifier and both data from game level 0 and level 2 were used for the input features.

^b^Differences were statistically significant at *P*<.05.

## Discussion

### Principal Results

By comparing the classifiers and games, we learned that the game focusing on speed was more suitable for predicting the motor skill level than the game focusing on precision. Data obtained from playing the roadrunner game led to significantly better performances than data obtained from playing the maze game. Thus, adding the game focusing on precision did not improve our preliminary results. The important contribution of the roadrunner data to the classification performance may be explained by the fact that speed is an important component in the MABC-2 as well. Two out of 3 subtests of the fine MABC-2 are time-sensitive. These findings correspond to the results of Rivera et al [18], who showed that the time for completing a task was an important component for intraindividual variability with their tested sensor-augmented toy.

By comparing the types of data and the LoDs, we learned that the combination of both sensor and game features was the most suitable for predicting the motor skill level. For the combination of data obtained from playing level 0 and level 2, using both sensor and game features led to a significantly better performance than only using sensor features. Although the contribution of the sensor features to the performance was shown to be little, the addition of the gyroscope data led to improved results compared to our preliminary results [9]. The significant difference between using both sensor and game features and only using sensor features for the best performing combination of levels is interesting. This means that the game component of the assessment approach is not only beneficial for playfulness but it also plays an important role in the prediction of the motor skill level itself together with the sensor data. An interesting follow-up project could be to generate additional game features or design more speed-based games and study how they affect the prediction of the fine MABC-2 score. The fact that no significant differences were found between the DT classifier and the other 3 classifiers indicates that the selection of input features has more impact on the performance than the selection of the classifier. Although the DT classifier did not perform significantly better, it is preferred over the KNN and SVM algorithms since it gives insight into the classification process. This is an important characteristic for the teachers who will use the toy in their classrooms.

### Strengths, Limitations, and Opportunities

The best achieved accuracy of 0.76 and F1 score of 0.70 on predicting the label of the fine MABC-2 are promising for assessing children’s motor skills with sensor-augmented toys. Since the cube is easy to use in the classroom, it is relatively easy to collect data. Therefore, the current approach might not only be useful for one-time assessment but could also be used for monitoring. Although we predicted the outcome of the fine MABC-2 and this assessment indicates whether children have fine motor development problems or not, we cannot state that we can predict children’s fine motor skill level with playing games with the Futuro Cube. To do so, we should repeat our research and replace the fine MABC-2 labels with expert view labels of the motor skill level. Moreover, assessment with the Futuro Cube only indicates whether children might have fine motor development problems in general. However, fine motor development is complex and consists of several aspects. Important factors are, for instance, cognitive ability, anticipatory control, motor planning function, and spatial ability [4]. Since all of these factors are included in the tasks of the fine MABC-2, we included them in the games of the Futuro Cube as well. Thus, our toy only signals motor development problems in general but does not assess specific aspects of motor development. In case the assessment tool showed that a child was likely to have fine motor development problems, follow-up examination is required to investigate individual aspects or causes of fine motor development problems.

The current approach might not only be useful for assessment but might also be useful as a first screening tool. In that case, children who are not likely to have motor problems are already being filtered out with the results of the game. A valid and reliably fine motor skill assessment test such as the fine MABC-2 can be taken for the children who were likely to have fine motor skill problems based on the game results. That way, not all children have to take the fine MABC-2 test, and the false positives of the assessment with the toy can be filtered out afterwards.

Another promising opportunity of the Futuro Cube is using it for training instead of monitoring. Developing a valid and reliable assessment toy will take some time, but playing with the Futuro Cube might also be useful for training purposes. Children could train their fine motor skills while playing games with the toy. This could be valuable for both children with motor skill problems without having specific disorders as well as children with, for instance, cerebral palsy or fine motor problems after a stroke. For learning, fun is an important factor since it improves intrinsic motivation and focus [25]. It is shown that gamified training is highly engaging and boosts the motivation of players [12]. Therefore, such a playful way of training their motor skills would be a valuable addition to the current methods. When the toy is ready for assessment, it could also be used to monitor progress in therapy or rehabilitation of such children. Since data are wirelessly sent in real time to a computer, such training opportunities could be improved by making the game adaptive. The level could be fitted to the child’s capacities, which improves the attention span and motivation. In this study, we focused on predicting the outcome of the fine MABC-2, but we did not include feasibility and usability in our approach. Although we did not study playfulness for children and usability for teachers in our approach, both children and teachers were very enthusiastic and their informal responses were, without exception, positive.

### Conclusions

This study examined the possibilities of using sensor-augmented toys to assess children’s fine motor skills. Such toys are less time-consuming and more playful and motivating than the current assessment methods. Compared to our preliminary research, we added the gyroscope for extra sensor data and an extra game that focused on precision instead of speed. With the best achieved accuracy of 0.76 and F1 score of 0.70, we showed that sensor-augmented toys can efficiently predict the outcome of the fine MABC-2 score. The selection of features is more important for the performance than the selection of the machine learning classifier. Classifiers that used input features obtained from playing the game focusing on speed performed significantly better that classifiers that used input features obtained from playing the game focusing on precision. Although our findings are a good start, further research is needed to develop a reliable and valid playful assessment tool. Possible improvements may be generating more game features, designing more speed-based games, and making the LoDs adaptive. Such adaptive games may also be valuable for training or rehabilitation purposes.

## Abbreviations

DT: decision tree
KNN: k-nearest neighbor
LoD: level of difficulty
LED: light-emitting diode
LR: logistic regression
MABC-2: Movement Assessment Battery for Children Second Edition
SVM: support vector machine
